# Unveiling the Bioactive Potential of *Allium colchicifolium* Boiss Bulb Flavonoids: Anti-cancer Activities, and Computational Exploration of Anti-angiogenic Mechanisms

**DOI:** 10.5812/ijpr-163152

**Published:** 2025-10-01

**Authors:** Mohammad Bagher Majnooni, Mustafa Ghanadian, Kamran Mansouri, Golam Reza Bahrami, Mahdi Mojarrab

**Affiliations:** 1 Pharmaceutical Sciences Research Center, Health Institute, Kermanshah University of Medical Sciences, Kermanshah, Iran; 2 Department of Pharmacognosy, Isfahan Pharmaceutical Sciences Research Center, School of Pharmacy and Pharmaceutical Sciences, Isfahan University of Medical Sciences, Isfahan, Iran; 3 Medical Biology Research Center, Health Technology Institute, Kermanshah University of Medical Sciences, Kermanshah, Iran

**Keywords:** *Allium colchicifolium*, Phytochemical Study, Flavonoids, Cytotoxicity, Anti-angiogenesis, VEGF, Column Chromatography, Molecular Docking

## Abstract

**Background:**

Plants of the genus *Allium* show significant anti-cancer properties due to various phytochemicals, including flavonoids.

**Objectives:**

This study investigated the cytotoxic activities of the methanolic extract of *Allium colchicifolium* bulbs and its purified flavonoids. It also assessed the anti-angiogenic activities, a key mechanism of anti-cancer agents.

**Methods:**

The methanolic extract was fractionated using column chromatography (CC) on silica gel RP-18 and polyamide SC-6, then purified with Sephadex LH-20. The compounds were identified through spectroscopic methods such as nuclear magnetic resonance (NMR) and liquid chromatography-electrospray ionization-tandem mass spectrometry (LC-ESI-MS/MS). Cytotoxicity and anti-angiogenic activities were evaluated using the MTT assay and the chick chorioallantoic membrane (CAM) assay, respectively. Computational modeling explored the potential anti-angiogenic mechanisms of the purified compounds. Additionally, absorption, distribution, metabolism, excretion, and toxicity (ADMET) profiling predicted drug-likeness features.

**Results:**

Three flavonoids — quercetin 3-O-rutinoside (1), isorhamnetin 3-O-glucoside (2), and quercetin (3) — were isolated and identified. Compounds 2 and 3 showed the highest cytotoxicity against PC3 (prostate cancer, IC_50_ = 1.72 ± 0.11 µg/mL) and MCF-7 (breast cancer, IC_50_ = 1.64 ± 0.11 µg/mL) cell lines. The methanolic extract and compound 3 also had potent anti-angiogenic effects with IC_50_ values of 4.2 ± 0.25 and 5.3 ± 0.3 µg/mL, respectively. Molecular docking indicated that compounds 1 and 3 had the strongest interactions with the vascular endothelial growth factor receptor (VEGFR)-1, consistent with their anti-angiogenic activity. The ADMET profiling showed that compound 3 had the highest similarity to drug-like molecules.

**Conclusions:**

This was the first phytochemical study of flavonoids in *A. colchicifolium* bulbs. The results suggest that these bulbs could serve as a natural source for cancer prevention and treatment, owing to their cytotoxic and anti-angiogenic properties. Further research is needed to confirm these findings, and in vivo studies are essential to validate their therapeutic potential.

## 1. Background

Flavonoids are a key group of polyphenolic compounds characterized by a C6-C3-C6 carbon structure, and their anti-cancer properties have long been a focus of research (1). Flavonoids prevent the proliferation and growth of cancer cells by blocking cellular signaling pathways involved in apoptosis and cell cycle regulation, such as mitogen-activated protein kinases (MAPK), phosphoinositide 3-kinase (PI3K)/protein kinase B (AKT)/mammalian target of rapamycin (mTOR), nuclear factor-kB (NF-kB), Janus kinases (JAK)/signal transducer and activator of transcription (JAK/STAT3), among others (1-3). Additionally, flavonoids exhibit prominent anti-angiogenic effects by inhibiting the expression of genes that mediate angiogenesis, including vascular endothelial growth factor (VEGF) and hypoxia-inducible factor-1 alpha (HIF-1α), thereby preventing tumor growth and metastasis (4). Clinical studies have shown that flavonoids improve the likelihood of treating various cancers. Besides impacting the proliferation, growth, migration, and metastasis of tumor cells, these compounds also increase the cells’ sensitivity to chemotherapy and help reduce drug resistance (1, 5).

*Allium* species (Amaryllidaceae), including *Allium flavum* (1), *A. ascalonicum* (2), *A. cepa* (3), *A. sativum*, and *A. fistulosum* (4), exhibit prominent anti-cancer and anti-angiogenic activities due to their diverse secondary metabolites. These include flavonoids such as quercetin and kaempferol, as well as organosulfur compounds like diallyl thiosulfinate (allicin) and diallyl sulfides (5-7). Abdel-Hady et al. demonstrated that a kaempferol derivatives-rich extract of *A. kurrat* exhibited cytotoxic activities against colon and liver cancer cell lines (8). Additionally, a quercetin-rich fraction from *A. cepa* revealed cytotoxic activities against adrenal carcinoma cell lines (3). A flavonoid-rich extract of *A. hirtifolium* prevented vessel growth in various in vitro angiogenesis models by blocking VEGF gene expression (9). An in vivo study on *A. flavum* and *A. carinatum* extracts exhibited that these high-flavonoid extracts, both alone and in combination with the broad-spectrum chemotherapeutic drug doxorubicin, significantly inhibited angiogenesis (10).

The promising anticancer effects of flavonoids such as hesperidin, quercetin, apigenin, and luteolin, and their glycosylated derivatives, have led to the design and implementation of numerous clinical trials across various phases for these compounds (11).

*Allium colchicifolium* (synonym: *A. bischoffii* Hausskn. ex Dinsm) is one of the *Allium* species native to western Iran, particularly the mountains of Kermanshah province. This plant was first documented by Wendelbo in 1971 on Bisotun Mountain, east of Kermanshah, Iran (12, 13). In folk medicine, *A. colchicifolium* is used to treat rheumatoid arthritis, joint pain, cholesterolemia, and inflammation. It is also used to prepare several local foods (14, 15).

## 2. Objectives

Based on this evidence, the present study purified flavonoids from the methanol (MeOH) extract of *A. colchicifolium* bulbs and identified them using one-dimensional and two-dimensional nuclear magnetic resonance (NMR) spectroscopy and liquid chromatography-electrospray ionization-tandem mass spectrometry (LC-ESI-MS/MS). We also evaluated the cytotoxic and anti-angiogenic activities of the extract and its isolated flavonoids.

## 3. Methods

### 3.1. General Experimental Procedures

A Bruker Avance AV 400 ^1^H NMR [400 MHz, DMSO-d6 (99.9% purity, Mesbah Energy, Iran)] and ^13^C NMR [100 MHz, DMSO-d6 (99.9% purity, Mesbah Energy, Iran)] were used to record NMR spectra. The heteronuclear multiple bond correlation (HMBC) NMR was used to determine two and three-bond heteronuclear ^1^H-^13^C connectivity. An Agilent LC (1200 series, Germany)-ESI-MS/MS (Agilent Technologies, Palo Alto, CA, USA) was used for molecular ion mass analysis. The column chromatography (CC) was run on polyamide-SC6 (Roth, Germany) and Sephadex-LH (Pharmacia Fine Chemicals, Sweden) and eluted with gradient solvent systems, chloroform (CHCl_3_): MeOH (Merck, Germany). Thin-layer chromatography (TLC) was run on TLC silica gel (SiO_2_, Merck, Germany) with CHCl_3_: MeOH 9:1. 1% natural product reagent (NP, 2-aminoethyl diphenylborinate, Merck, Germany) was used to visualize polyphenol spots on TLC. All statistical analyses were performed using SPSS software version 23.0 and Excel 2016.

### 3.2. Plant Material

*Allium colchicifolium* was gathered from Mian Rehan (34°35′02″N 47°26′34″E) in Kermanshah province, western Iran, during the flowering period from April to May by local herbal collectors trained by herbarium experts at the Kermanshah Faculty of Pharmacy. This plant was confirmed by comparison with the voucher specimen (No. 001-036-001-160) available in the herbarium of the Pharmacognosy Department, Faculty of Pharmacy, Kermanshah University of Medical Sciences (16).

### 3.3. Extraction and Isolation

The *A. colchicifolium* dried bulbs (350 g) were ground and extracted sequentially with hexane, CHCl_3_, CHCl_3_/MeOH (9:1), and MeOH solvents (1.5 L each, for 3 days) at room temperature using the method described by Fattorusso et al. (17). All extracts were filtered through Whatman No. 1 filter paper and concentrated with a rotary evaporator (50°C, 70 mbar). The MeOH extract (10 g) was chromatographed on a silica gel RP-18 column (3 × 30 cm) using a gradient solvent system from H_2_O to MeOH (100 → 0) to yield 10 fractions (Fr.1-Fr.10). The TLC profile (SiO_2_, CHCl_3_: MeOH, 9:1) of Fr.5 (H_2_O: MeOH, 50:50) and Fr.6 (H_2_O: MeOH, 40:60) showed most yellow spots after spraying the NP and were selected as rich-polyphenol fractions. Fr.5 and Fr.6 were re-chromatographed on a polyamide SC6 column (3 × 40 cm) using a linear gradient solvent system from CHCl_3_ to MeOH (95:5, 90:10, 85:15, 80:20, 70:30, each 500 mL). According to the TLC profile, Fr.5f (CHCl_3_ to MeOH 70:30), Fr.6c (CHCl_3_ to MeOH 85:15), and Fr.6d (CHCl_3_ to MeOH 80:20) were selected for the next steps. Fr.5f was chromatographed on a Sephadex LH-20 column (3 × 80 cm; MeOH) to yield Fr.5f1 and Fr.5f2. Fr.5f1 was obtained in a pure state as compound 1 (8 mg, Rf = 0.22). Fr.6c and Fr.6d were re-chromatographed under the same column conditions to yield compound 2 (9 mg, Rf = 0.3) and compound 3 (11 mg, Rf = 0.55), respectively.

### 3.4. Cytotoxicity Activities

#### 3.4.1. Cell Lines and Culture

Cancer cell lines, including PC3 NCBI-C427 (human prostate adenocarcinoma), HeLa NCBI-C115 (human cervix carcinoma), MCF7 NCBI-C135 (human breast adenocarcinoma), and a normal cell line, human umbilical vein endothelial cell (HUVEC) NCBI-C554, were purchased from the Pasteur Institute of Tehran, Iran. Cells were cultured and maintained in Dulbecco’s modified Eagle’s medium (DMEM) supplemented with 10% fetal bovine serum (FBS) and 2% (v/v) streptomycin-penicillin in a humidified incubator with a 5% CO_2_ atmosphere at 37°C. Passages 2 to 3 of the cells were utilized for the cytotoxicity assay (18).

#### 3.4.2. MTT Assay

The MTT assay was used to determine the cytotoxic activities of the MeOH extract, isolated compounds (1 - 3), and doxorubicin (as a positive control) on human cancer cell lines. For the MTT assay, concentrations (1, 5, 10, 25, 50, 100 µg) of the samples were prepared in DMEM as diluent and incubated with cells (10^5^ cells/mL) for 24 hours in 96-well plates. Then, the MTT powder was added to the wells and incubated for 4 hours at 37°C. Absorbance was measured at 570 nm using a microplate reader. The experiment was performed in triplicate, and cell viability percentages were calculated by the following equation: % Cell viability = [(OD negative control - OD tested compounds)/(OD negative control)] × 100. The negative control was untreated cells. The cytotoxicity IC_50_ value for the extracts was defined as the concentration that reduces 50% cell viability (19).

### 3.5. Anti-angiogenic Activities

The anti-angiogenesis activities of the MeOH extract and isolated compounds (1-3) were evaluated using the chicken chorioallantoic membrane (CAM) model. Briefly, pathogen-free fertilized chicken eggs were purchased from Baharan Parent Stock Company, Kermanshah, Iran, and incubated at 37°C with 75% humidity for 7 days. Then, square windows (1 cm^2^) were opened in the outer shell of the eggs, and different concentrations of samples were loaded. After 48 hours, the zones around the discs were photographed with a digital camera and macroscopically assayed for neovascular zones of CAM in each treatment group. The effects on local vessel density within a 10 cm^2^ area surrounding the windows were measured in each treatment group using AngioSoft software (V, 2019) (12, 13). The study was done in triplicate for each experimental group (8 eggs). The anti-angiogenesis IC_50_ value for the extracts was reported as the concentration that reduces 50% angiogenesis compared to the blank disk.

### 3.6. Molecular Docking

Crystal structures of the target proteins, VEGF-R1 [Protein Data Bank (PDB) ID: 3HNG]; VEGF-A (PDB ID: 1VPF), VEGF-B (PDB ID: 2C7W), VEGF-C (PDB ID: 2X1X), VEGF-D (PDB ID: 2XV7) were downloaded from the PDB (http://www.RCSB.org). The co-crystallized ligands, cofactors, and water molecules were deleted. Afterwards, hydrogen atoms were added and Gasteiger charges were assigned using Chimera 1.13 (14). Ligand structures generated by ACDLabs ChemSketch (freeware, 2015 2.5) (15) were prepared by adding hydrogens, Gasteiger charges, and energy minimization through Chimera 1.13. The active site of vascular endothelial growth factor receptor (VEGFR)-1 (PDB ID: 3HNG) was identified from its co-crystallographic ligand. For recognizing binding sites of VEGF-A, VEGF-B, and VEGF-D, the Computed Atlas of Surface Topography of Proteins (CASTp) server (http://sts.bioe.uic.edu/castp/) was utilized (20). In the case of VEGF-C (PDB ID: 2X1X), several residues at the interface of VEGF-C and VEGFR-2 were selected as the binding site (21). Molecular docking was conducted by Autodock Vina in PyRx 0.8 software with exhaustiveness of 50 for each target protein. The grid box dimensions for VEGFR-1 and VEGFR-2 were determined to be 28.41 × 28.02 × 27.79 Å and 28.25 × 25.00 × 22.20 Å, respectively, to cover the binding sites (22). Then, LigPlot^+^ (V 2.2) was used to analyze the type of interactions between protein and ligand (23).

### 3.7. Absorption, Distribution, Metabolism, Excretion, and Toxicity Analysis

The success of a drug is dependent on an acceptable absorption, distribution, metabolism, excretion, and toxicity (ADMET) profile in addition to the good efficacy of the drug (24). In this study, the physicochemical properties of three compounds were extracted using the ADMETlab 2.0 web tool, and Lipinski’s Rule of 5 (Ro5) was used for evaluating drug-likeness (25). Moreover, pharmacokinetic parameters and toxicity of these compounds were predicted by the AdmetSAR database (http://lmmd.ecust.edu.cn/admetsar1/).

## 4. Results and Discussion

### 4.1. Isolation and Identification of Compounds

**Figure 1.**
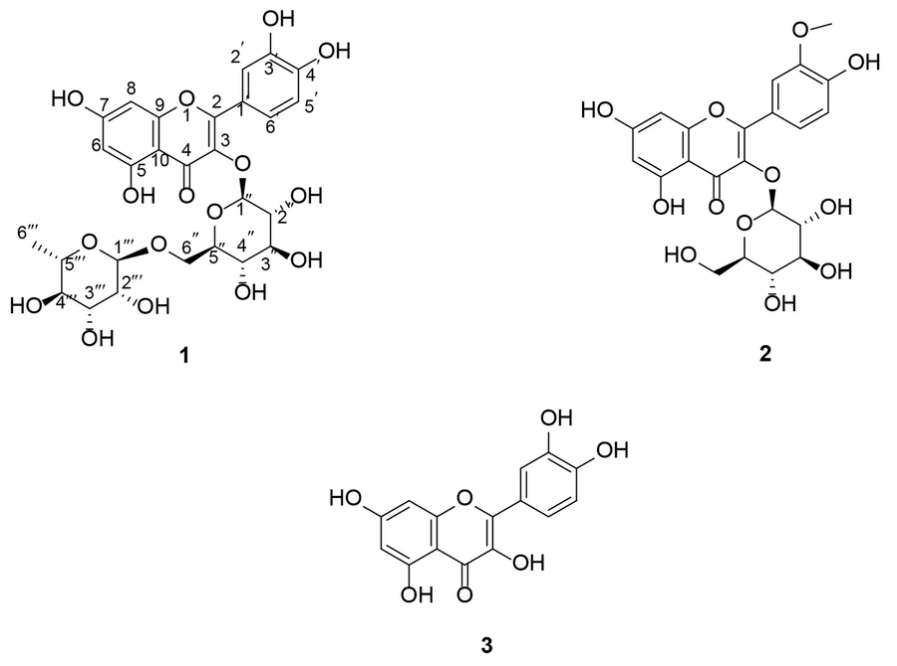
Chemical structure of flavonoids isolated from *Allium colchicifolium* bulbs

Plants and their active compounds have long been of interest to researchers for cancer treatment. In this study, we investigated the phytochemical profile of *A. colchicifolium* bulbs along with their cytotoxic and anti-angiogenic activities. The *A. colchicifolium* dried bulbs (350 g) were successfully extracted with hexane, CHCl_3_, CHCl_3_: MeOH (9:1), and MeOH solvents, with yields of 3.5 g (1%), 5.6 g (1.6%), 17.5 g (5%), and 14.7 g (4.2%), respectively. MeOH was fractionated and purified using CC on RP-18, SC6 polyamide, and Sephadex LH-20, which yielded seven fractions and three purified flavonoids (Figure 1), including quercetin 3-O-rutinoside (compound 1), isorhamnetin 3-O-glucoside (compound 2), and quercetin (compound 3).

Based on the LC-MS/MS analysis in negative mode and collision energy 15 eV, compound (1) exhibited a molecular ion [M-H]^-^ at 609 m/z. The results of fragmentation of 609 m/z showed 463 m/z, 301 m/z, 273 m/z, and 151 m/z, which corresponded to a glycosylated flavonoid (Appendix 1 in Supplementary File) (26, 27). Additionally, the ^1^H NMR spectra of (1) showed five protons in the aromatic region at δH 6.19 (1H, d, J = 2.1 Hz, H-6), 6.38 (1H, d, J = 2.1 Hz, H-8), 6.92 (1H, d, J = 8.48 Hz, H-5ʹ), 6.84 (1H, d, J = 8.3 Hz, H-5ʹ), 7.53 (1H, d, J = 2.3 Hz, H-2ʹ), 7.55 (1H, dd, J = 8.3, 2.3 Hz, H-6ʹ). Also, two signals at δH 5.4 (1H, d, J = 7.30 Hz, H-1ʹʹ) and 4.45 (1H, bs, H-1ʹʹʹ) corresponded to anomeric protons of glucose and rhamnose, respectively. Protons of glucosyl H-2ʺ-H-6ʺ and rhamnosyl H-2ʺʹ-H-5ʺʹ overlapped at 3.12-5.2 ppm, and rhamnosyl H-6ʺʹ appeared at 1.11 (1H, d, J = 6.4 Hz). The signals of OH groups appeared at δH 12.66 (1H, s, 5-OH), 10.91 (1H, s, 7-OH), 9.75 (1H, s, 3ʹ-OH), and 9.26 (1H, s, 4ʹ-OH). Besides, the ^13^C NMR of compound (1) showed 27 carbon signals. The signals at δc 100.72 and 101.13 corresponded to the anomeric carbon of rhamnose and glucose, respectively. The carbon signal of C-6ʹʹʹ and C-4 (carbonyl group) appeared at δc 17.79 and 177.32.

Additionally, the HMBC analysis revealed the correlation between the H anomeric of glucose with C-3 and the H anomeric of rhamnose with H-6ʹʹ. Other HMBC analysis results were depicted in Appendix 2 in Supplementary File. According to the aforementioned information, compound 1 was identified as quercetin 3-O-rutinoside (rutin). Rutin was isolated from *A. cepa* and several other *Allium* species (7, 28, 29).

The results of fragmentation of compound 2 by LC-MS/MS showed the molecular ions at [M-H]⁻ 477 m/z, [M-H-Glu] 314 and 315 m/z, [315-OH] 299 m/z, [315-OCH₃] 284 m/z, [299-CO] 271 m/z, and [284-C_8_H_6_O_2_] 151 m/z, which confirmed a methoxy glucosylated flavonoid structure for compound 2 (26, 27). On the other hand, the presence of five protons in aromatic regions (δH 6 - 8 ppm), a signal at δH 3.85 (3H, s, 3ʹ-OCH_3_), a doublet signal corresponding to the anomeric H of glucose at δH 5.58 (1H, d, J = 7.26 Hz), and the presence of 22 carbons in ^13^C NMR analysis confirmed the isorhamnetin 3-O-glucoside structure for compound 2 (30). *Allium* macrostemon and A. neapolitanum are other sources of isorhamnetin 3-O-glucoside (31, 32). Also, our team isolated isorhamnetin 3-O-glucoside from leaves of *A. colchicifolium* in a parallel study (16).

Based on the LC-MS/MS analysis in negative mode, compound 3 showed a molecular ion [M-H]^-^ at 301 m/z. The fragmentation results of 301 m/z produced [M-H-CH_3_] 284 m/z, [M-H-CO] 271 m/z, [M-H-CH_3_-CO] 255 m/z, and [M-H-CH_3_-C_8_H_6_O_2_] 151 m/z, confirming the flavonoid structure of compound 3. Also, the ^1^H NMR and ^13^C NMR spectra of compound 3 corresponded to quercetin isolated from *A. colchicifolium* leaves in our previous study, so compound 3 was identified as quercetin (20).

Compound 1: Yellow powder, ^1^H-NMR in DMSO-d6 (400 MHz) ppm, δH: 1.11 (1H, d, J = 6.4 Hz, H-6ʺʹ), 3.12-5.2 (overlapped, H-2ʺ-H-6ʺ and H-2ʺʹ-H-5ʺʹ), 4.45 (1H, bs, H-1ʹʹʹ), 5.4 (1H, d, J = 7.30 Hz, H-1ʹʹ), 6.19 (1H, d, J = 2.1 Hz, H-6), 6.38 (1H, d, J = 2.1 Hz, H-8), 6.92 (1H, d, J = 8.48 Hz, H-5ʹ), 6.84 (1H, d, J = 8.3 Hz, H-5ʹ), 7.53 (1H, d, J = 2.3 Hz, H-2ʹ), 7.55 (1H, dd, J = 8.3, 2.3 Hz, H-6ʹ), 9.26 (1H, s, 4ʹ-OH), 9.75 (1H, s, 3ʹ-OH), 10.91 (1H, s, 7-OH), 12.67 (1H, s, 5-OH); ^13^C-NMR (100 MHz, DMSO-d6) δc: 177.32 (C-4), 164.04 (C-7), 161.19 (C-5), 156.59 (C-2), 156.52 (C-9), 148.38 (C-3ʹ), 144.72 (C-4ʹ), 133.26 (C-3), 121.56 (C-6ʹ), 121.13 (C-1ʹ), 116.15 (C-5ʹ), 115.19 (C-2ʹ), 103.93 (C-10), 101.13 (C-1ʹʹ), 100.72 (C-1ʹʹʹ), 98.64 (C-6), 93.56 (C-8), 76.39 (C-3″), 75.86 (C-5″), 74.03 (C-2″), 71.79 (C-4ʹʹʹ), 70.51 (C-3ʹʹʹ), 70.33 (C-2ʹʹʹ), 69.95 (C-4″), 68.21 (C-5ʹʹʹ), 66.96 (C-6″), 17.79 (C-6ʹʹʹ). Negative ESI mass (m/z): (M-H) – 609, 463, 301, 285, and 150.9.

Compound 2: Yellow powder, ^1^H-NMR in DMSO-d6 (400 MHz) ppm, δH: 3.11 - 3.58 (overlapped, H-2″- H-6″), 3.85 (3H, s, 3ʹ-OCH_3_), 5.58 (1H, d, J = 7.10, H-1ʹʹ), 6.21 (1H, d, J = 2.14, H-6), 6.42 (1H, d, J = 2.14, H-8), 6.92 (1H, d, J = 8.37, H-5ʹ), 7.50 (1H, dd, J = 8.37, 2.05, H-6ʹ), 7.95 (1H, d, J = 2.05, H-2ʹ), 9.89 (1H, s, 4ʹ-OH), 12.61 (1H, s, 5-OH); ^13^C-NMR (100 MHz, DMSO-d6) δc: 177.35 (C-4), 164.57 (C-7), 161.19 (C-5), 156.40 (C-2), 156.21 (C-9), 149.37 (C-3ʹ), 146.85 (C-4ʹ), 132.89 (C-3), 121.99 (C-6ʹ), 121.04 (C-1ʹ), 115.17 (C-5ʹ), 113.42 (C-2ʹ), 103.91 (C-10), 100.72 (C-1ʹʹ), 98.76 (C-6), 93.71 (C-8), 77.41 (C-5″), 76.36 (C-3″), 74.30 (C-2″), 69.75 (C-4″), 60.53 (C-6″), and 55.61 (3ʹ–OCH_3_). Negative ESI mass (m/z): (M-H) − 477, 315, 314, 299, 284, 271, and 151.

Compound 3: Pale yellow powder, ^1^H-NMR in DMSO-d6 (400 MHz) ppm, δH: 6.20 (1H, d, J = 1.75 Hz, H-6), 6.43 (1H, d, J = 1.75 Hz, H-8), 6.91 (1H, d, J = 9.92 Hz, H-5ʹ), 7.56 (1H, dd, J = 9.92, 1.7 Hz, H-6ʹ), 7.70 (1H, d, J = 1.7 Hz, H-2ʹ), 12.52 (1H, bs, 5-OH); ^13^C-NMR (100 MHz, DMSO-d6) δc: 175.52 (C-4), 164.45 (C-7), 160.45 (C-5), 156.15 (C-9), 147.77 (C-4ʹ), 146.65 (C-2), 145.09 (C-3ʹ), 135.64 (C-3), 121.88 (C-6ʹ), 119.92 (C-1ʹ), 115.61 (C-5ʹ), 115.00 (C-2ʹ), 102.75 (C-10), 98.30 (C-6), and 93.39 (C-8). Negative ESI mass (m/z): (M-H) - 301 m/z, 284 m/z, 271 m/z, 255 m/z, and 151 m/z.

### 4.2. Cytotoxicity Activities

The cytotoxic activities of isolated flavonoids and the MeOH extract are presented in Table 1. Compound 2 demonstrated significant cytotoxic activity against PC3 (P < 0.01), while compound 3 exhibited significant cytotoxic activity against MCF-7 and HeLa (P < 0.01), compared to other samples. Additionally, compound 3 and the MeOH extract exhibited the highest cytotoxic activity on HUVEC (P < 0.01), compared to the other isolated compounds. Several studies have shown the cytotoxic effects of flavonoids, including those isolated from *Allium* plants (6, 7). A noteworthy point in these studies is the lower cytotoxic effects of flavonoids on normal cell lines compared to cancer cells, which may indicate fewer side effects of flavonoids (33). Xiao and Parkin demonstrated the cytotoxic effects of *A. cepa* and its phenolic compounds on liver cancer lines (34).

**Table 1.** Cytotoxic Activities of Isolated Compounds (1-3) and MeOH Extract of *Allium colchicifolium* Bulbs ^a, b^

Variables	PC3	MCF-7	HeLa	HUVEC
**Compound 1**	2.96 ± 0.16	3.16 ± 0.3	6.95 ± 0.32	9.35 ± 0.8
**Compound 2 **	1.72 ± 0.02 ^c^	2.58 ± 0.21	8.43 ± 0.32	9.96 ± 0.37
**Compound 3**	3.11 ± 0.1	1.64 ± 0.11 ^d^	6.17 ± 0.13 ^e^	7.23 ± 0.29 ^f^
**MeOH extract**	2.16 ± 0.02 ^c^	1.84 ± 0.06 ^d^	5.39 ± 0.33 ^e^	6.83 ± 0.21 ^f^
**Doxorubicin **	0.051 ± 0.004 ^g^	0.034 ± 0.01 ^g^	0.066 ± 0.007 ^g^	0.045 ± 0.006 ^g^

Abbreviation: HUVEC, human umbilical vein endothelial cell.

^a^ Values are expressed as IC_50_ (µg/mL) and mean ± SD.

^b^ The results of the cytotoxic activity of each compound were compared with the other compounds, and the statistical power was calculated to be 95%.

^c^ Significant difference in cytotoxic activities on PC3 (post-hoc Tukey, P < 0.01).

^d^ Significant difference in cytotoxic activities on MCF-7 (post-hoc Tukey, P < 0.01).

^e^ Significant difference in cytotoxic activities on HeLa (post-hoc Tukey, P < 0.01).

^f^ Significant difference in cytotoxic activities on HUVEC (post-hoc Tukey, P < 0.01).

^g^ Significant difference in cytotoxic activities on the study cell lines (post-hoc Tukey, P < 0.001).

### 4.3. Anti-angiogenic Activities

**Figure 2.**
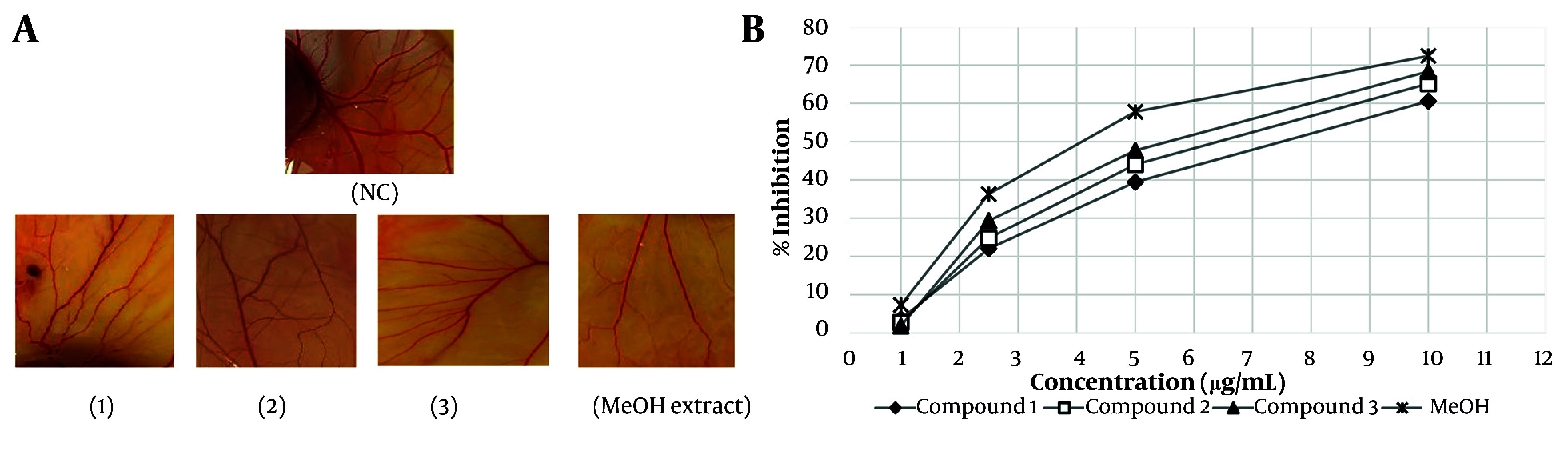
A, anti-angiogenesis activities of isolated compounds (1-3) and the MeOH extract of *Allium colchicifolium* bulbs on the chorioallantoic membrane (CAM) model of angiogenesis at 10 µg/mL; B, percentage of neovascular inhibition of isolated compounds (1-3) and the MeOH extract from *A. colchicifolium* bulbs (abbreviation: NC, negative control).

The results of the anti-angiogenesis activities of isolated flavonoids (1 - 3) and the MeOH extract of *A. colchicifolium* bulbs on the CAM model of angiogenesis are shown in Figure 2A and B. The IC_50_ of each compound was considered as the concentration that caused 50% inhibition of angiogenesis in a 100 mm^2^ area surrounding each sample disc compared to the negative control disc (distilled water). The MeOH extract (IC_50_ = 4.2 ± 0.25 µg/mL, P < 0.001) and compound 3 (5.3 ± 0.3 µg/mL, P < 0.01) revealed significant inhibitory effects on neovascularization compared to compounds 1 (IC_50_ = 7.12 ± 0.1 µg/mL) and 2 (IC_50_ = 6 ± 0.21 µg/mL). Quercetin and its derivatives have shown anti-angiogenesis activities in various studies. Tan and co-workers demonstrated that quercetin at 10 nM on the CAM model exhibited anti-angiogenesis by blocking matrix metalloproteinase-2 gene expression (35). Inhibition of protein kinase C (PKC), VEGF, and AKT/mTOR are among other mechanisms of anti-angiogenesis by quercetin derivatives (36-38). Alonso-Castro et al. showed that rutin (compound 1) inhibited angiogenesis at 20 mg/kg by reducing mice VEGF serum levels (39). Additionally, quercetin and 8-methylquercetin-3,5,7,3',4'-pentamethyl ether demonstrated anti-angiogenesis activities at 25 µM through blocking VEGF receptor-2 and angiogenesis cellular signaling pathways (40). In a meta-analysis study, Khater et al. showed that the presence of sugar groups in position 3 has no significant effect on the anti-angiogenic activities of flavonoids, which was also observed in our study (41).

### 4.4. Molecular Docking

**Figure 3.**
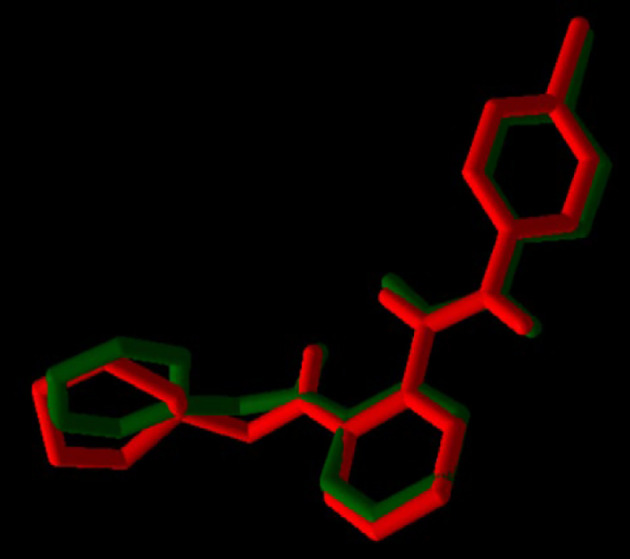
Validation of docking protocol; co-crystallized ligand (8ST): Green; redocked ligand: Red.

**Figure 4.**
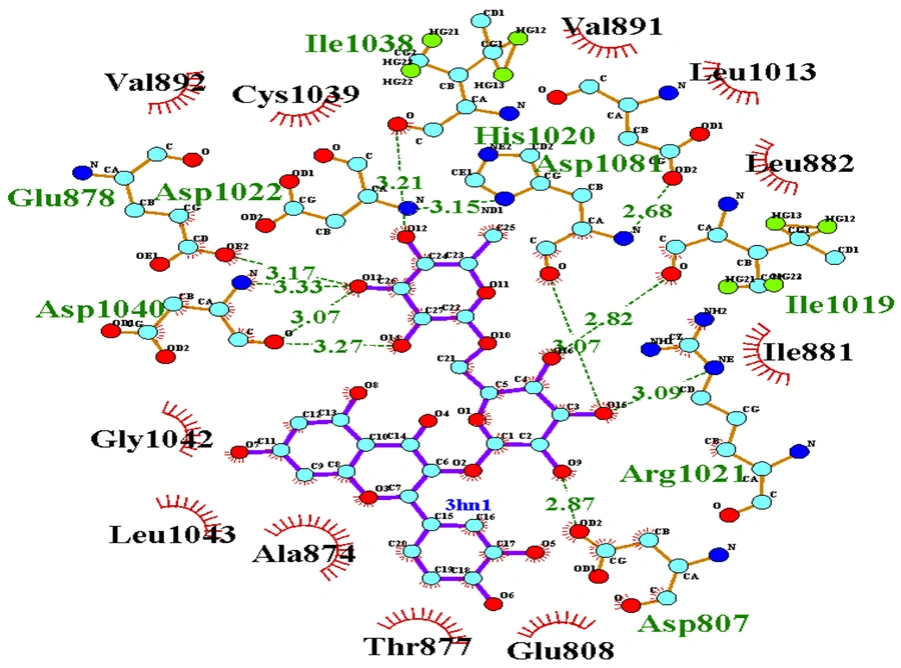
2D Interaction of compound 1 with vascular endothelial growth factor (VEGF)-R1: Compound 1 formed hydrogen bonds with Asp1040 (3.27, 3.07, and 3.33 Å), Glu878 (3.17 Å), and Ile1038 (3.21 Å) and hydrophobically interacted with Leu882 and Cys1039.

For validation of docking, the co-crystallographic ligand (8ST) within VEGF-R1 (PDB ID: 3HNG) was redocked. As shown in Figure 3, 8ST and its redocked structure overlapped well. In the present study, the RMSD value was 0.44, which was within the range of < 2 .0 Å, indicating the accuracy of the docking protocol (42). Binding affinities of compounds on protein targets are shown in Table 2. Compounds 1 (Figure 4) and 3 showed the highest interaction with VEGF-R1 (-9.7 kcal/mol), and compound 3 showed the highest interaction with VEGF-A, C, and D, with binding affinities of -9.4, -6.6, and -7.2 kcal/mol, respectively. The strong interaction of compound 3 with VEGF-R1 and VEGFs can justify the stronger activities of this compound compared to other compounds in the CAM model (Figure 2). 

**Table 2.** Binding Affinity Values Against the Target Proteins

Ligand	VEGF-R1 (3HNG)	VEGF-A (1VPF)	VEGF-B (2C7W)	VEGF-C (2X1X)	VEGF-D (2XV7)
**Compound 1**	-9.7	-7.5	-7.2	-5.7	-6.2
**Compound 2**	-8.2	-7.9	-7.0	-5.8	-6.1
**Compound 3**	-9.7	-9.4	-7.0	-6.6	-7.2

Abbreviation: VEGF, vascular endothelial growth factor receptor.

### 4.5. Absorption, Distribution, Metabolism, Excretion, and Toxicity Analysis

The Lipinski Ro5 is commonly used for evaluating drug-likeness, which states that absorption or permeation is more likely for compounds possessing a molecular weight (MW) < 500, lipophilicity (LogP) < 5, a number of hydrogen bond acceptors (HBA) (sum of Ns and Os) < 10, and a number of hydrogen bond donors (HBD) (sum of OHs and NHs) < 5. At most one violation is acceptable (43). Compound 3, based on Ro5, is drug-like (Table 3). The topological polar surface area (TPSA) values for compound 3 were less than 140 Å^2^, indicating proper permeability in the cell membrane (44). In terms of pharmacokinetic properties, compounds 1, 2, and 3 failed blood-brain barrier (BBB) permeation (Table 4). All compounds are human intestinal absorption (HIA^+^) and P-glycoprotein (P-gp) substrates. Additionally, only compound 3 may be an inhibitor of CYP2C9. All these compounds are non-carcinogenic and are weak inhibitors of hERG. The hERG encodes a voltage-gated potassium channel in cardiac myocytes that plays an important role in action potential membrane repolarization, and hERG channel blockade is associated with long QT syndrome (45). Overall, compound 3 exhibited better ADMET profiles compared to others, although in terms of mutagenicity (46), compound 3 showed positive AMES toxicity.

**Table 3.** Drug-Likeness Prediction Through ADMETlab

Ligand	MW (≤ 500)	HBA (≤ 10)	HBD (≤ 5)	LogP	TPSA (Å^2^)	Lipinski Rule
**Compound 1**	610.150	16	10	-0.038	269.430	Rejected
**Compound 2**	478.110	12	7	0.559	199.510	Rejected
**Compound 3**	300.070	5	3	3.678	90.900	Accepted

Abbreviations: MW, molecular weight; HBA, hydrogen bond acceptors; HBD, hydrogen bond donors; TPSA, topological polar surface area.

**Table 4.** Pharmacokinetics and Toxicity Prediction by AdmetSAR

Ligand	BBB Permeant	GI Absorption	P-gp Substrate	CYP2C9 Inhibitor	hERG Inhibition	Carcinogens	AMES Toxicity
**Compound 1**	BBB-	HIA^+^	Substrate	Non-inhibitor	Weak inhibitor	Non-carcinogenic	Non-AMES toxic
**Compound 2**	BBB-	HIA^+^	Substrate	Non-inhibitor	Weak inhibitor	Non-carcinogenic	Non-AMES toxic
**Compound 3 **	BBB-	HIA^+^	Substrate	Inhibitor	Weak inhibitor	Non-carcinogenic	AMES toxic

Abbreviations: GI, gastrointestinal; HIA, human intestinal absorption; BBB, blood-brain barrier; P-gp, P-glycoprotein; CYP, cytochrome P450; hERG, human ether-a-go-go-related gene.

### 4.6. Conclusions

In this study, two glycosylated flavonoids, including rutin (compound 1) and isorhamnetin 3-O-glucoside (compound 2), and one aglycone, including quercetin (compound 3), were isolated by CC methods and identified from *A. colchicifolium* bulbs for the first time. Additionally, the cytotoxic and anti-angiogenic activities of the MeOH extract of the *A. colchicifolium* bulbs and its isolated flavonoids were investigated using MTT and CAM models, respectively. The results of the present study demonstrated the notable cytotoxic and anti-angiogenic activities of the MeOH extract. Furthermore, compound 3, as an aglycone flavonoid, exhibited significant anti-angiogenesis activities with an IC_50_ of 5.3 ± 0.3 µg/mL and cytotoxic effects with an IC_50_ ≤ 3 µg/mL against PC3 and MCF, which is consistent with other studies (40). Additionally, a docking study assessed the interaction of the compounds with VEGF, one of the most critical mediators of angiogenesis induction (47, 48), and compound 3 exhibited the strongest interaction. The anti-cancer effects of edible plants and their phytochemicals, including A. plants and their flavonoids, as reliable and available sources, have always attracted the attention of researchers to discover effective molecules for the treatment of various types of malignancy (40, 41). On the other hand, the crucial limitation of using flavonoids for the treatment of various diseases, including malignancies, is their low bioavailability. The use of new drug delivery techniques to increase their bioavailability offers a potential solution. Furthermore, experimental studies have shown that flavonoids can have synergistic effects with chemotherapy drugs. This synergy could enable a reduction in the dosage of chemotherapy drugs with high side effects, such as doxorubicin, in patients (49). According to our results, the MeOH extract of *A. colchicifolium* can be a rich source of polyphenolic compounds, including anticancer flavonoids. Our previous study on the leaves of this plant also showed the presence of polyphenolic compounds with antibacterial and biofilm activities. Therefore, considering the aforementioned biological effects, *A. colchicifolium* can be introduced as a promising candidate for drug molecule discovery (16). Therefore, comprehensive clinical trials are suggested to assess their bioavailability and efficacy.

## supplementary material

ijpr-24-1-163152-s001.pdf

## Data Availability

The dataset presented in the study is available on request from the corresponding author during submission or after publication.
