# Supplementary figures and images for: Unveiling the Bioactive Potential of Allium colchicifolium Boiss Bulb Flavonoids: Anti-cancer Activities, and Computational Exploration of Anti-angiogenic Mechanisms
Source: Iran J Pharm Res. 2025 Oct 1;24(1):e163152. doi: 10.5812/ijpr-163152 (PMC12523642; doi:10.5812/ijpr-163152)

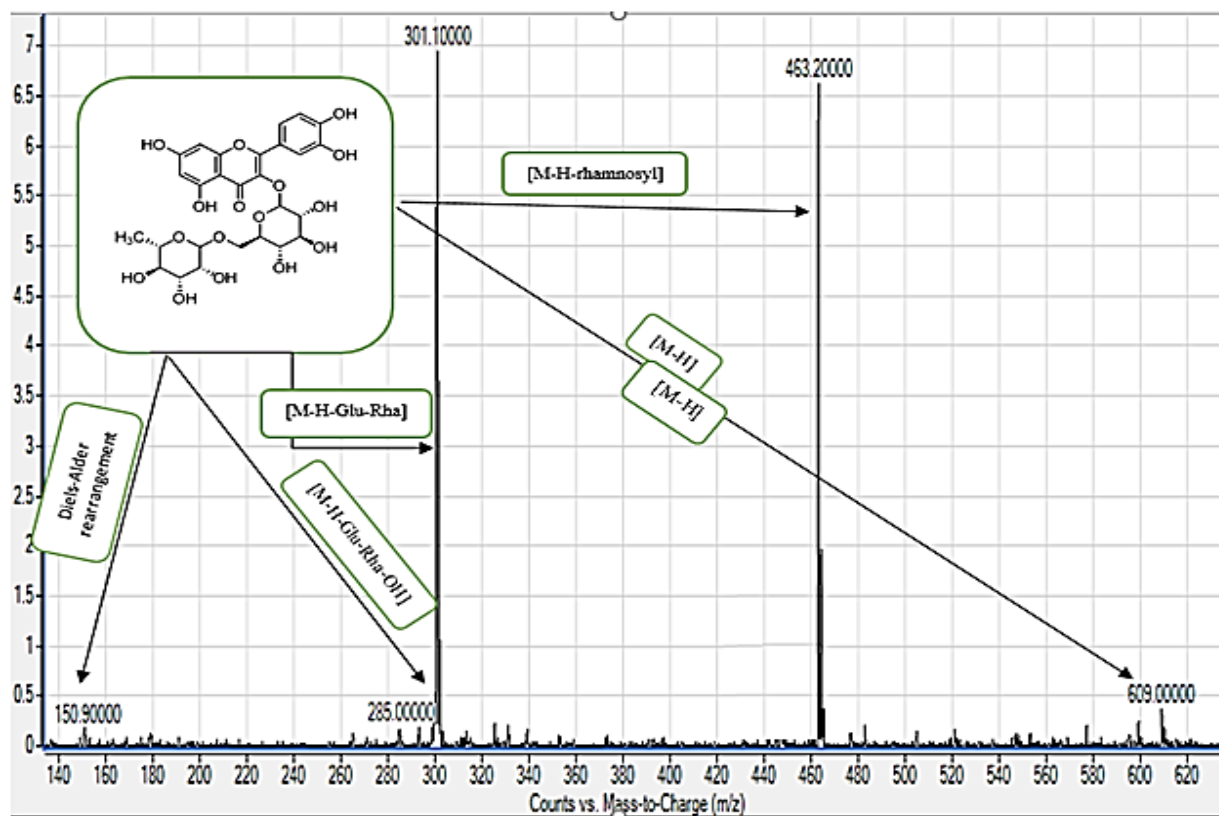

**Appendix 1.** LC-MS/MS analysis of compound 1

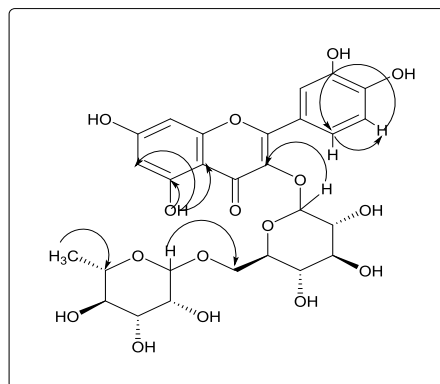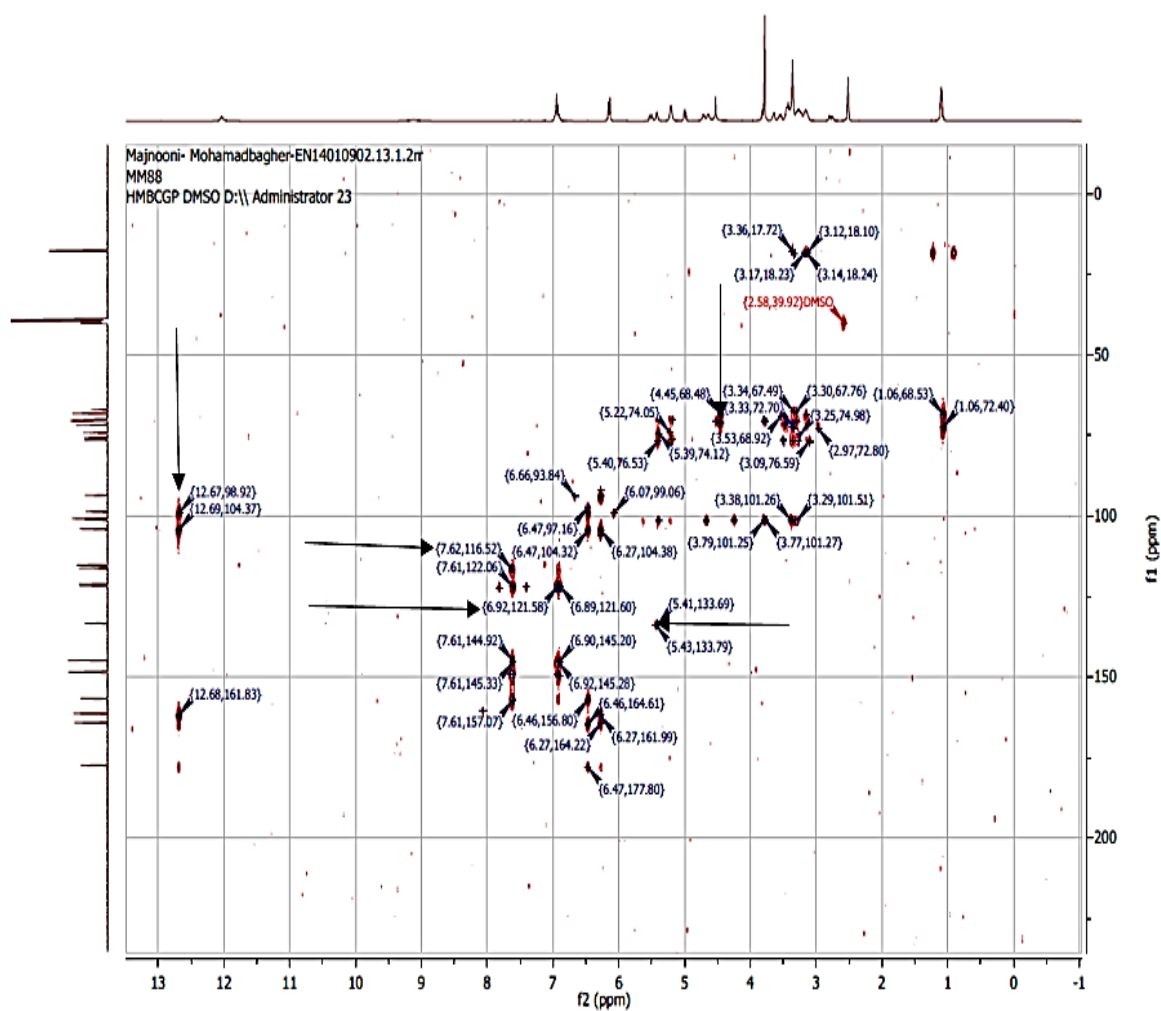

**Appendix 2.** Key HMBC correlations of compound 1

Supplement: ijpr-24-1-163152-s001.pdf [file ijpr-24-1-163152-s001.pdf]
